# Enzymatic characterization of a recombinant carbonyl reductase from *Acetobacter* sp. CCTCC M209061

**DOI:** 10.1186/s40643-017-0169-1

**Published:** 2017-08-28

**Authors:** Ping Wei, Yu-Han Cui, Min-Hua Zong, Pei Xu, Jian Zhou, Wen-Yong Lou

**Affiliations:** 1 Lab of Applied Biocatalysis, School of Food Science and Engineering, South China University of Technology, Guangzhou, 510640 Guangdong China; 2 School of Chemistry and Chemical Engineering, South China University of Technology, Guangzhou, 510640 Guangdong China

**Keywords:** Carbonyl reductase, *Acetobacter* sp., Chiral alcohols, Enzymatic characterization

## Abstract

**Background:**

*Acetobacter* sp. CCTCC M209061 could catalyze carbonyl compounds to chiral alcohols following anti-Prelog rule with excellent enantioselectivity. Therefore, the enzymatic characterization of carbonyl reductase (CR) from *Acetobacter* sp. CCTCC M209061 needs to be investigated.

**Results:**

A CR from *Acetobacter* sp. CCTCC M209061 (AcCR) was cloned and expressed in *E. coli*. AcCR was purified and characterized, finding that AcCR as a dual coenzyme-dependent short-chain dehydrogenase/reductase (SDR) was more preferred to NADH for biocatalytic reactions. The AcCR was activated and stable when the temperature was under 35 °C and the pH range was from 6.0 to 8.0 for the reduction of 4′-chloroacetophenone with NADH as coenzyme, and the optimal temperature and pH were 45 °C and 8.5, respectively, for the oxidation reaction of isopropanol with NAD^+^. The enzyme showed moderate thermostability with half-lives of 25.75 h at 35 °C and 13.93 h at 45 °C, respectively. Moreover, the AcCR has broad substrate specificity to a range of ketones and ketoesters, and could catalyze to produce chiral alcohol with *e.e.* >99% for the majority of tested substrates following the anti-Prelog rule.

**Conclusions:**

The recombinant AcCR exhibited excellent enantioselectivity, broad substrate spectrum, and highly stereoselective anti-Prelog reduction of prochiral ketones. These results suggest that AcCR is a powerful catalyst for the production of anti-Prelog alcohols.

**Graphical abstract:**
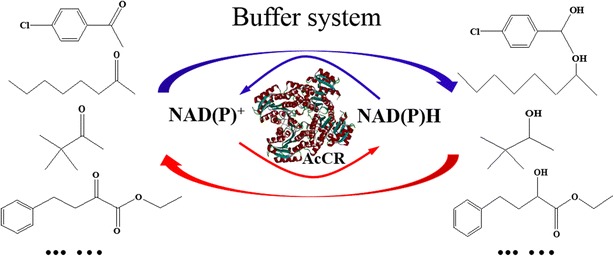
The biocatalytic reactions conducted with the recombinant AcCR

## Background

Chiral alcohols are vitally important building blocks for the synthesis of pharmaceuticals, agricultural chemicals, flavors, and special materials. Currently, both chemical and biological methods can be used to synthesize chiral alcohols. The most prospective biocatalytic method is the asymmetric transfer of hydrogenation to carbonyl groups catalyzed by reductases or microbial cells containing relevant reductases (Li et al. 2014; Zheng et al. 2017). In recent years, the efficient asymmetric reduction of ketones using biocatalysts as its high yield and excellent enantiomeric excess has been extensively used for the production of chiral alcohols in the chemical and pharmaceutical industries (Birolli et al. 2015; Gutierrez et al. 2010).

Carbonyl reductase (CR) or alcohol dehydrogenase (ADH) is NAD(P)H-dependent oxidoreductase which can catalyze a variety of carbonyl compounds to the corresponding alcohols (Kaluzna et al. 2005). Enantioselective CRs or ADHs have been reported from animals (Nakajin et al. 1998; Montfort et al. 2002), plants (Sengupta et al. 2015), and microorganisms (Grosch et al. 2015; Singh et al. 2009). A number of CRs or ADHs have been utilized for asymmetrically reducing carbonyl functionalities, and many excellent biocatalytic processes have been developed (Li et al. 2015). Among all the sources of CRs, microorganisms are the most important providers, and some CRs or ADHs from microorganisms have been reported with high yield and enantioselectivity (Musa and Phillips 2011). The carbonyl reductase SCRII from *Candida parapsilosis* could catalyze the asymmetric reduction of 2-hydroxyacetophenone to (*S*)-1-phenyl-1,2-ethanediol with optical purity of 100% in high yield of 98.1% (Zhang et al. 2011). Xu et al. (2015) found that an NADPH-dependent carbonyl reductase from *Yarrowia lipolytica* ACA-DC 50109 could efficiently convert α-chloroacetophenone to (*R*)-2-chloro-1-phenylethol with 99% *e.e.* Ni et al. (2011) heterologously overexpressed a *β*-ketoacyl-ACP reductase from *Bacillus* sp. ECU0013 in *E. coli*, which was used for efficient reduction of ethyl 2-oxo-4-phenylbutyrate at 620 g/L, and the *e.e.* of the product ethyl (*S*)-2-hydroxy-4-phenylbutyrate was excellent (>99%). Therefore, using carbonyl reductases to catalyze the asymmetric reduction of prochiral carbonyl compounds is an efficient and useful method for the synthesis of chiral alcohols (He et al. 2016; Cui et al. 2017; Qian et al. 2014).

The oxidoreductase from *Acetobacter* is mostly used to produce acetic acid, and rarely used to asymmetric reduction of carbonyl compounds to chiral alcohols. In our previous study, it was found that *Acetobacter* sp. CCTCC M209061 could catalyze the asymmetric reductions of several carbonyl compounds to corresponding chiral alcohols following anti-Prelog rule with excellent enantioselectivity (Cheng et al. 2014; Wei et al. 2015, 2016). In the present work, the AcCR was heterologously expressed and systematically characterized for its substrate spectrum, stereoselectivity, and the capacity of industrial application.

## Methods

### Chemicals, bacterial strains, and plasmids

The *Acetobacter* sp. CCTCC M209061 strain was previously isolated by our group from Chinese kefir grains and stored at −80 °C. *E. coli* DH5α, BL21(DE3)pLysS, and plasmid pGEX-2T were purchased from Novagen. The restriction enzyme FastDigest AvaI, EcoRI, T4 DNA Ligase, DNA, and protein marker were purchased from Thermo Scientific. KOD FX polymerase for PCR was purchased from Toyobo. The kits used in the construction of the recombinant plasmids were purchased from Generay. The prochiral ketones were purchased from Sigma Aldrich or Aladdin. The primers synthesis and DNA sequencing were completed by Sangon Biotech. All other reagents and solvents were of analytical grade and used without further purification.

### Expression of AcCR in *E. coli* BL21(DE3)pLysS

Genomic DNA of *Acetobacter* sp. CCTCC M209061 was extracted and purified using a bacterial genomic DNA Kit. Oligonucleotide primers for *accr* were designed according to the published gene sequence of oxidoreductase from *Acetobacter pasteurianus* 386B (Sequence ID: HF677570.1). The DNA fragment of *accr* was amplified with primer 1 (5′-TCCCCCGGGAATGGCACGTGTAGCAGGCAAGGTT-3′) and primer 2 (5′-CCGGAATTCCTTATTGCGCGGTGTACCCACCATCAAT-3′) and double-digested with *Ava*I and *Eco*RI, then the *accr* fragment was inserted into vector pGEX-2T, the resulting plasmid (pGEX-*accr*) was transformed into *E. coli* DH5α for amplification, and then the correct pGEX-*accr* was transformed into *E. coli* BL21(DE3)pLysS to express the recombinant AcCR. The recombinant *E. coli* BL21(DE3)pLysS(AcCR) were cultivated at 37 °C, 180 rpm in 50 mL LB medium (pH 6.5) containing 100 μg/mL of ampicillin. When the optical density at 600 nm (OD_600_) of the culture reached 1.2, the temperature was changed to 20 °C, and then IPTG was added to a final concentration of 0.4 mM. The cultivation continued at 20 °C for additional 15 h. Then the recombinant cells were harvested by centrifugation (8000 rpm, 5 min) at 4 °C and washed three times with physiological saline (0.85%), and stored at 4 °C for later use.

### Measurement of enzymatic activity

The oxidordeuctase activity of the recombinant AcCR was determined by monitoring the change of the absorbance at 340 nm for 3 min on the spectrophotometer (Shinmadzu UV-3010, Japan). The AcCR-catalyzed reduction of 4′-chloroacetophenone was conducted with NADH or NADPH at 35 °C in 2 mL phosphate buffer (pH 6.5 or 5.5). One unit (U) of recombinant AcCR was defined as the amount of enzyme that catalyzed the 1 μmol NADH or NADPH per minute. The NAD^+^- and NADP^+^-linked oxidations catalyzed by AcCR were conducted with isopropanol as substrate and NAD^+^ or NADP^+^ at 45 °C in 2 mL 50 mM citrate–phosphate buffer (pH 8.0). One unit (U) of AcCR was defined as the amount of enzyme that catalyzed the production of 1 μmol NADH or NADPH per minute. The reaction solution was incubated at 35 or 45 °C for 3 min, and then 20 μL recombinant AcCR (about 0.008 mg of the purified enzyme) was added to proceed the reaction.

### Purification of recombinant AcCR

The harvested recombinant *E. coli* BL21(DE3)pLysS(AcCR) cells were suspended in 50 mM citrate–phosphate buffer (pH 6.5) at the concentration of 50 mg/mL, ultrasonicated for 3 × 18 min, and then the cells debris was removed by centrifugation at 12,000 rpm at 4 °C for 20 min. The resulting supernatant was filtered with 0.22-μm filter membrane before the purification process. The general step of the purification using NGC Quest™ 10 system was as follows. First, the GST-based affinity chromatography column (5 mL) was pre-equilibrated using buffer A (4.3 mM Na_2_HPO_4_, 1.47 mM KH_2_PO_4_, 137 mM NaCl, 2.7 mM KCl, pH7.3), and then the crude enzyme was loaded on the column. After that, the loaded column was first subjected to washing with 10 column volume buffer A to remove the unbound protein fractions, then washed sequentially with buffer B (added 0.5 M NaCl in buffer A) to remove some stubborn protein, and then buffer A was used once again to lower the salinity. At last, the target protein was eluted with buffer C (50 mM Tris–HCl, 2.5 g/L glutathione, pH 8.0). The protein content was measured by the method of Bradford (1976).

### Enzymatic characteristic of the recombinant AcCR

#### Effect of temperature

The effects of temperature on the activity of recombinant AcCR were determined at various temperatures from 20 to 55 °C. For thermal stability determination, the enzyme was pre-incubated at various temperatures ranging from 20 to 55 °C, equivalent was taken at a certain time, and the residual activity was determined as described in “Measurement of enzymatic activity.” The coenzyme NADH and NAD^+^ was used for the reduction of 4′-chloroacetophenone (5 mM) and the oxidation of the isopropanol (150 mM), respectively. The enzyme activity of the first measurement was defined as 100%.

#### Effect of pH

The optimum pH of recombinant AcCR was investigated within a pH range of 4.5–9.5 using various buffer systems at 50 mM. The buffers were as follows: citrate–phosphate (pH 4.5–8.0), Tris–HCl (pH 8.0–8.5) and glycine–NaOH (pH 8.6-9.5). The pH stability was evaluated by pre-incubating the recombinant AcCR in different pH buffers (4.5–9.5) at 4 °C for 4 days. Samples were taken at a certain time, and the residual activity was determined as described above with the non-incubated recombinant AcCR as the control.

#### Effect of metal ions and chemical agents

The influence of metal ions and chemical agents on the catalytic activity of recombinant AcCR was investigated by pre-incubating the enzyme with various additives in citrate–phosphate buffer (50 mM, pH 6.5) at 35 °C for 30 min. The enzyme activity was determined under the condition described above using NADH as coenzyme. The enzyme activity of the recombinant AcCR in the absence of additives was recorded as 100%.

#### Substrate specificity and bioconversion of various carbonyl compounds

The activity of recombinant AcCR for each specific substrate was measured by the method described above using NADH as the coenzyme. The relative activity of recombinant AcCR to 4′-chloroacetophenone was defined as 100%.

The reduction of various carbonyl compounds were performed in a 10-mL conical flask with 4 mL citrate–phosphate buffer (50 mM, pH 6.5) containing 50 mM each substrate, 0.1 mM NADH, and 150 mM isopropanol. The reactant was pre-incubated in a shaker at 35 °C for 10 min, and then 8 U recombinant AcCR was added to initiate the reaction. Samples (25 μL) were withdrawn after reaction for a certain time, and then the product and residual substrate were extracted with ethyl acetate (2 × 25 μL, 5 mM *n*-dodecane as internal standard) before GC analysis.

### Kinetic parameters assays

For the kinetic analysis, the initial reaction rates of the recombinant AcCR were determined under the optimum conditions. For the reduction of 4′-chloroacetophenone, the coenzyme NADH or NADPH varied from 0.05 to 0.6 mM or from 0.05 to 1.0 mM, and the concentrations of 4′-chloroacetophenone were from 0.5 to 10 mM. For the oxidation of isopropanol, the concentrations of NAD^+^ or NADP^+^ varied from 0.05 to 2 mM or from 0.5 to 20 mM. The isopropanol concentrations were from 30 to 275 mM. All the measurements were carried out in duplicate. Michaelis–Menten was used to fit the data, and the kinetic parameters of recombinant AcCR-catalyzed reduction and oxidation reactions, *K*_m_ and *V*_max_ values, were obtained in the fit before computing the *K*_cat_.

### GC methods

The organic extracts of reaction mixtures were analyzed by a GC analysis (Shimadzu Corp GC 2010). GC was performed using chiral columns HP Chiral 10B and CP-Chiralsil-Dex-CB. The initial reaction rate, the yield, and the product *e.e.* of those reactions were calculated as described in our previous report (Wang et al. 2013).

## Results and discussion

### Sequence analysis of AcCR

**Fig. 1:**
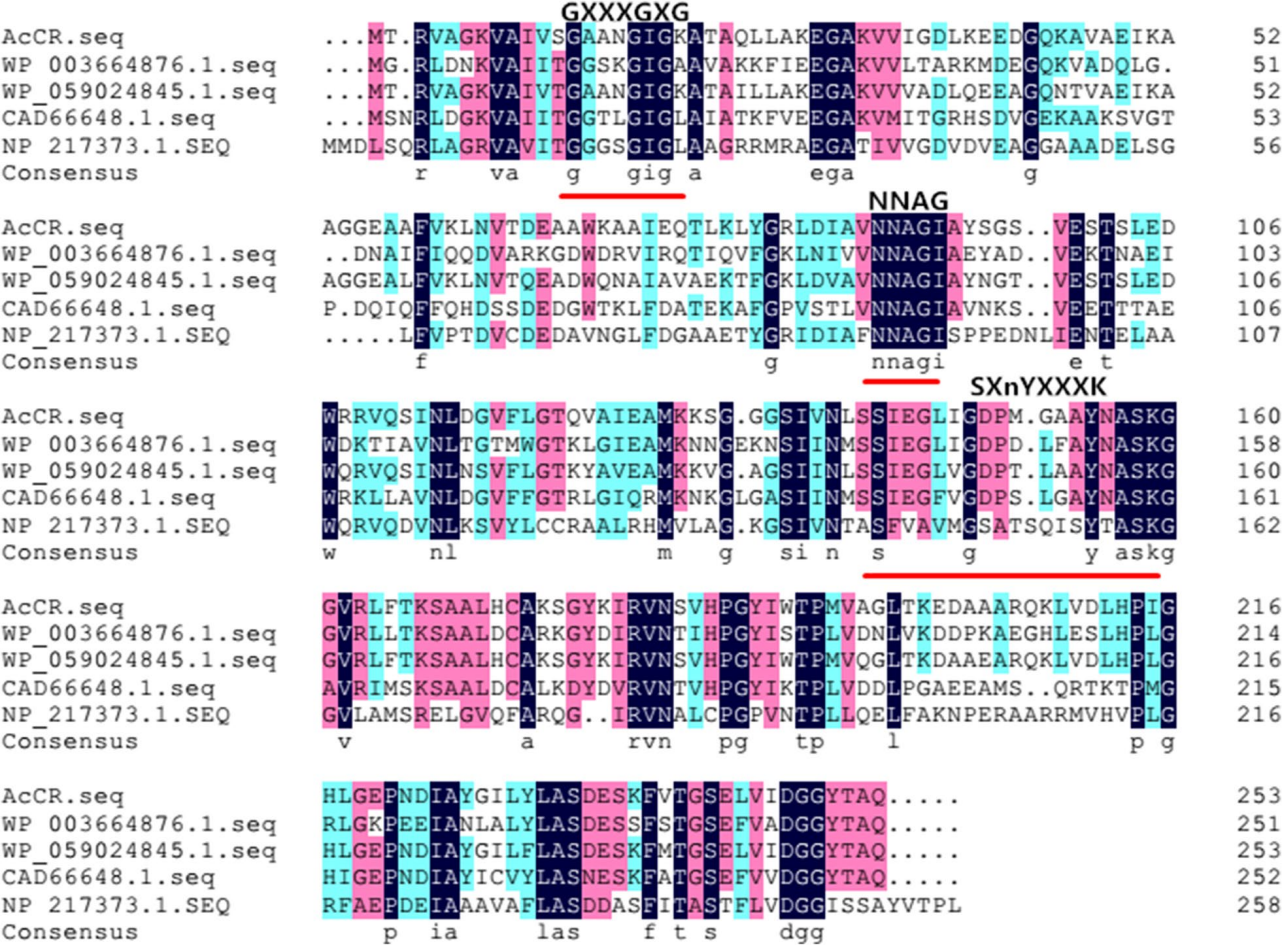
Alignment of multiple deduced amino acid sequences of AcCR and carbonyl reductases from other sources

A 762-bp polynucleotide sequence was amplified by PCR from the genome DNA of *Acetobacter* sp. CCTCC M209061. The sequence was an intact open reading frame and encoded a predicted protein of 253 amino acid residues with a molecular weight of about 26.4 kDa. The GenBank accession number of the nucleotide sequence of AcCR gene is MF419650. The result of multiple sequence alignment with NCBI protein blast is shown in Fig. 1. The AcCR amino acid sequence displayed a high level of similarity to the identical proteins from other *Acetobacter* and related bacteria. The identities of AcCR with other proteins were as follows: 84% with 3-beta hydroxysteroid dehydrogenase from *Acetobacter ghanensis* (WP_059024845.1), 56% with Cyclopentanol dehydrogenase from *Mesorhizobium plurifarium* (CDX57396.1), 51% with *R*-specific alcohol dehydrogenase from *Lactobacillus brevis* (CAD66648.1), and 37% with short-chain type dehydrogenase/reductase from *Mycobacterium tuberculosis* H37Rv (NP_217373.1), respectively. The amino acid sequence alignments of the deduced polypeptides of AcCR with the short-chain dehydrogenase/reductase (SDR) proteins from GenBank database were performed. The conserved sequence (GXXXGXG) (Rossman fold motif) (Masud et al. 2011; Jörnvall et al. 2010) reported importantly of the coenzyme binding site of SDR family enzyme was found at the N-terminal 13–19 position of the AcCR. And the common active site conserved amino acid pattern SXnYXXXK was emerged at the mid-chain pattern 142–159, as well as the NNAG (89–92) sequence which has a function in stabilizing β-strands of classical SDR (Persson et al. 2003; Filling et al. 2002). Therefore, the AcCR could be classified as the SDR which belongs to a bulky dehydrogenase/reductase family.

### Expression and purification of recombinant AcCR

**Fig. 2:**
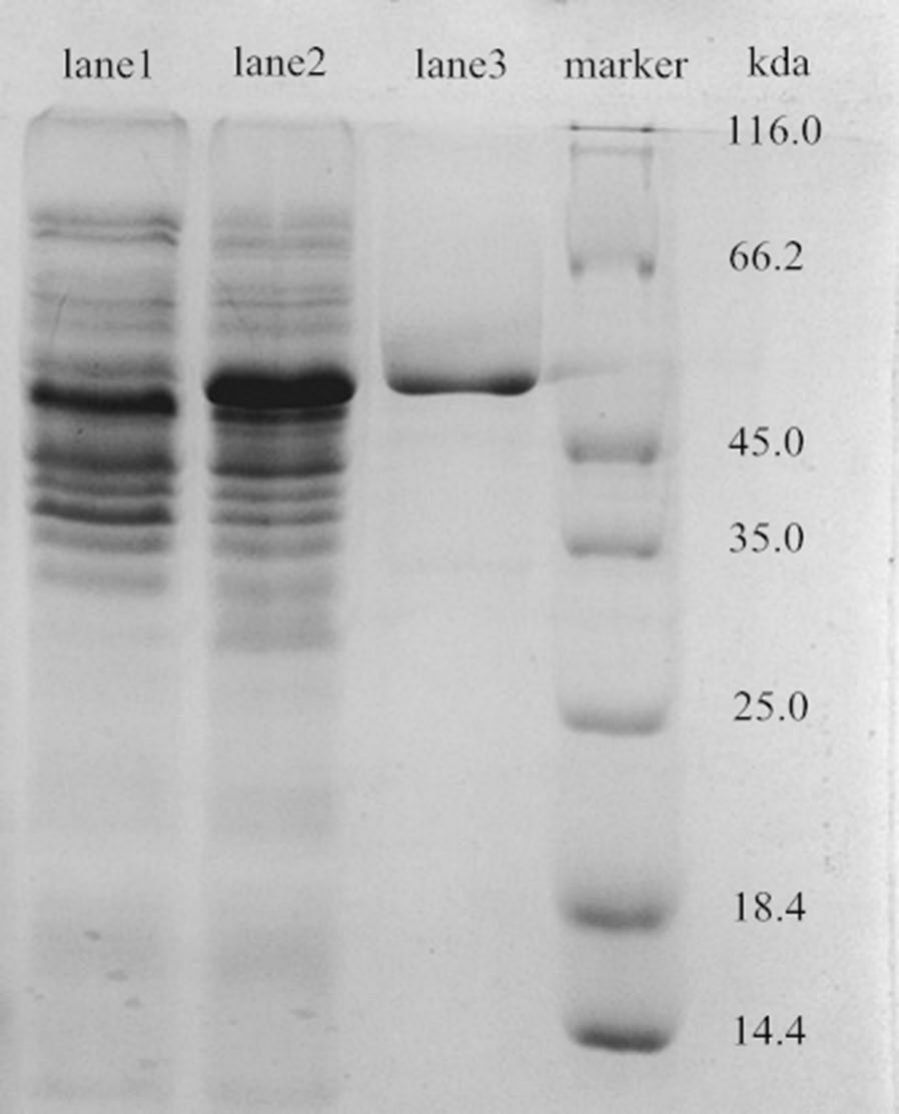
SDS-PAGE analysis of expression products by *E. coli* BL21 (DE3) pLysS(pGEX-*accr*): *lane 1* crude cell extract of non-IPTG-induced recombinant strain; *lane 2* crude cell extract of IPTG-induced recombinant strain; *lane 3* the purified recombinant AcCR

The *accr* was constructed into plasmid pGEX-2T and transformed into *E. coli.* BL21(DE3)pLysS. SDS-PAGE analysis of the crude extracts indicated that the recombinant AcCR was successfully expressed in the soluble form with the GST tag (shown in Fig. 2). As a GST-tagged fusion protein, the GAcCR (recombinant AcCR) was purified by a single-step affinity chromatography using a GST-tagged column. As expected, the purified GAcCR was observed at the position of approximately 53 kDa (the GST tag is about 26 kDa) which was consistent with the estimated value (Fig. 2). The purified recombinant AcCR activity was 5.17 U/mg and stored at 4 °C for later use.

### Enzymatic characteristic of the recombinant AcCR

#### Coenzyme preference of recombinant AcCR

**Fig. 3:**
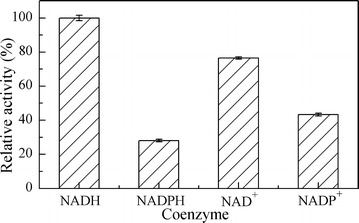
Coenzyme preference of the recombinant AcCR. Reaction conditions: 0.25 mM NADH or NADPH (0.5 mM NAD^+^ or NADP^+^), 2 mL 50 mM citrate–phosphate buffer (pH 6.5 for the reduction or pH 8.0 for oxidation), and 5 mM 4′-chloroacetophenone (150 mM isopropanol) were incubated for 5 min at 35 °C before adding 20 μL purified recombinant AcCR (about 0.008 mg of the purified enzyme), and then the changes of absorbance for 3 min were recorded

SDRs act on the substrate by transferring electrons from or to coenzyme. The recombinant AcCR was found to be a kind of carbonyl reductase which could use both NAD(H) and NADP(H) as coenzyme to execute the redox reactions. The coenzyme preference of the recombinant AcCR was examined by measuring enzyme activity using 4′-chloroacetophenone or isopropanol as substrate. As shown in Fig. 3, the recombinant AcCR showed redox activity with both NAD(H) and NADP(H) as coenzymes. Meanwhile, the relative activity of the recombinant AcCR with NADPH as coenzyme was only about 40% of that with NADH, which confirmed that AcCR exhibited coenzyme specificity for NADH over NADPH. In the recent reports, many biocatalysts catalyzing asymmetric reductions utilize NADPH as hydrogen donor (Leuchs and Greiner 2011; Richter and Hummel 2011; Ma et al. 2013; Zhang et al. 2015). ADHs capable of utilizing NADH as the coenzyme outperformed NADPH-dependent ones, since NADH was more stable than NADPH under operational conditions (Li et al. 2015) and economic than NADPH. The NAD(H)-preferred CRs could be advantageous for their application in industrial bioreduction system. Therefore, NAD(H) was used as the coenzyme of the enzymatic characteristic of the recombinant AcCR in the subsequent study.

#### Effect of temperature

**Fig. 4:**
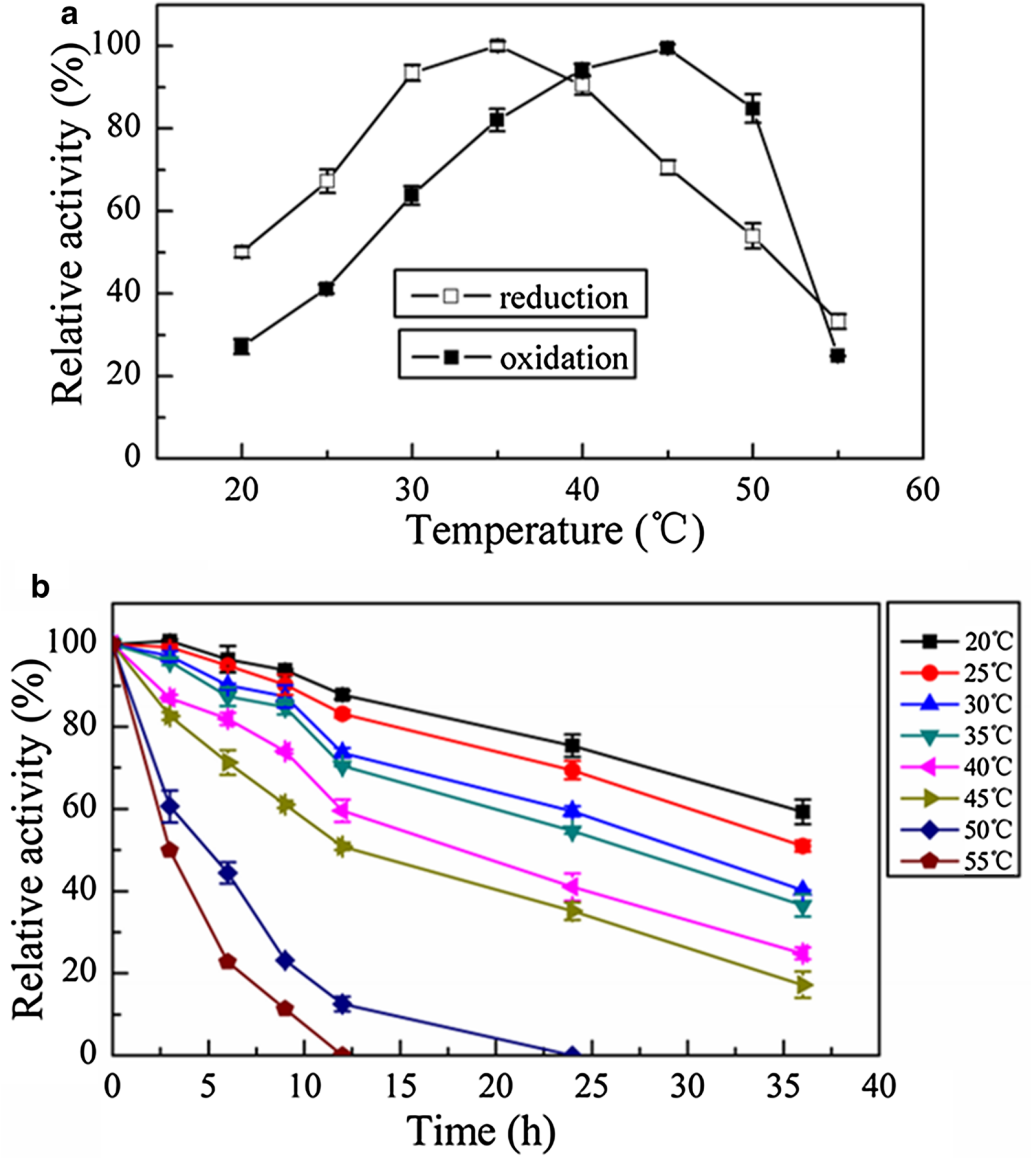
Effects of temperature on the activity and stability of the recombinant AcCR. Reaction conditions: **a** 0.25 mM NADH or 0.5 mM NAD^+^, 2 mL 50 mM citrate–phosphate buffer (pH 6.5 or 8.0), and 5 mM 4′-chloroacetophenone or 150 mM isopropanol were incubated at 20–55 °C for 5 min before adding 20 μL purified recombinant AcCR (about 0.008 mg of the purified enzyme), and the changes of absorbance for 3 min at 20–55 °C, respectively, were recorded; **b** 0.25 mM NADH, 2 mL 50 mM citrate–phosphate buffer (pH 6.5), and 5 mM 4′-chloroacetophenone were incubated for 5 min at 35 °C, then added 20 μL purified recombinant AcCR (about 0.008 mg of the purified enzyme, incubated at 20–55 °C), and the changes of absorbance for 3 min were recorded

Initially, the effect of varying temperatures on the activity of recombinant AcCR was explored on both reduction and oxidation reactions. The optimal temperature was found to be 35 °C for the reduction of 4′-chloroacetophenone when NADH was used as the coenzyme (Fig. 4a). For the case of the oxidation of isopropanol, the maximum activity was observed at 45 °C with NAD^+^ as coenzyme (Fig. 4a). The thermal stability of the recombinant AcCR was investigated at various temperatures ranging from 20 to 55 °C. As illustrated in Fig. 4b, the enzyme activity decreased slowly when the incubation temperature was less than 35 °C. The relative activity was over 50% after 36 h incubation at 30 °C, and even more than 62% after incubating for 24 h at 35 °C and over 5 h at 50 °C. Thus, the thermal stability of recombinant AcCR was excellent and much better than many reported carbonyl reductases from other sources (Xu et al. 2015; Singh et al. 2009; Luo et al. 2015; Wang et al. 2015). The enzyme exhibited half-lives of 25.75 h at 35 °C and 13.93 h at 45 °C, respectively, for the reduction and oxidation reactions.

#### Effect of pH

**Fig. 5:**
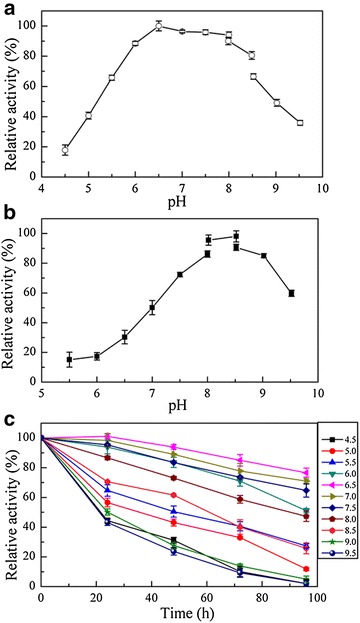
Effects of pH on the activity and stability of the recombinant AcCR. Reaction conditions: **a** 0.25 mM NADH, 2 mL 50 mM buffer (pH 4.5–9.5), and 5 mM 4′-chloroacetophenone were incubated at 35 °C for 5 min before adding 20 μL purified recombinant AcCR (about 0.008 mg of the purified enzyme), and then the changes of absorbance for 3 min at 35 °C were recorded; **b** 0.5 mM NAD^+^, 2 mL 50 mM buffer (pH 5.5–9.5), and 150 mM isopropanol were incubated at 45 °C for 5 min before adding 20 μL purified recombinant AcCR (about 0.008 mg of the purified enzyme), and then the changes of absorbance for 3 min at 45 °C were recorded; **c** 0.25 mM NADH, 2 mL 50 mM buffer (pH 4.5–9.5), and 5 mM 4′-chloroacetophenone were incubated for 5 min at 35 °C, then 20 μL purified recombinant AcCR was added (about 0.008 mg of the purified enzyme), and the changes of absorbance for 3 min were recorded

The effect of pH on recombinant AcCR was detected in different buffer systems (50 mM) at the range from 4.5 to 9.5 for both reduction and oxidation. The maximum activity of the recombinant AcCR for reduction of 4′-chloroacetophenone was obtained at pH 6.5. As shown in Fig. 5a, the relative activity was over 90% in the reduction reaction at the pHs between 6.0 and 8.0. The optimal pH for oxidation of isopropanol was found to be pH 8.5 when NAD^+^ was acted as coenzyme (Fig. 5b). The optimal pH of the recombinant AcCR for catalyzing reduction and oxidation is close to that of the oxidoreductase from *Gluconobacter oxydans* (Liu et al. 2014).

To investigate the pH stability, the recombinant AcCR was incubated at 4 °C in varying pHs from 4.5 to 9.5 and tested the reduction activity using NADH as coenzyme. As shown in Fig. 5c, the optimal storage pH was 6.5, at which pH almost 80% relative activity remained after 96 h. Almost 70% relative activities remained at both pH 7.0 and 7.5 after 96 h, as well as at pH 6.0 after 75 h. The results showed that the recombinant AcCR had broad pH tolerant scope and excellent pH stability.

#### Effect of metal ions and chemical agents

It is well known that metal ions have remarkable effects on the activity of carbonyl reductase. For example, Zn^2+^ is necessary for many medium-chain dehydrogenases/reductases (Zn-MDR). The Zn^2+^ appears in those enzymes to give the protein an additional strength, as if compensating for domain variability and evolutionary changes in the protein scaffold (Jörnvall et al. 2010). Besides, some metal ions also have negative effect on the activity of a number of enzymes. For example, Co^2+^, Cu^2+^, and Zn^2+^ can severely inhibit the enzyme activity of carbonyl reductase SCRII from *Candida parapsilosis* (Zhang et al. 2011). So it is of significantly important to explore the effects of various additives on the recombinant AcCR. As shown in Table 1, among the tested metal ions, Mn^2+^, K^+^, Fe^2+^, Mg^2+^, Ca^2+^, and Co^2+^ had slight stimulation on the recombinant AcCR, while the other metal ions had inhibitory effects to some extent. Enzyme activity was inhibited about 5 and 15% when 2 and 5 mM Zn^2+^ was added, and the residue enzyme activity was only 43.0 and 24.9% when 2 and 5 mM Cu^2+^ was added, respectively. Ag^+^ and Hg^+^ inhibited the enzyme activity completely.

**Table 1:** Effects of metal ions on the enzymatic activity of the recombinant AcCR

Reagent	Concentration (mM)	Relative activity (%)
Control		100.0 ± 0.8
Mn^2+^	2	122.3 ± 0.4
	5	119.9 ± 0.7
K^+^	2	110.6 ± 1.2
	5	103.4 ± 0.8
Fe^2+^	2	106.7 ± 1.0
	5	106.0 ± 2.9
Zn^2+^	2	94.8 ± 1.2
	5	85.8 ± 1.3
Mg^2+^	2	115.7 ± 0.7
	5	123.5 ± 2.4
Ba^2+^	2	100.8 ± 0.5
	5	97.9 ± 1.7
Ca^2+^	2	116.7 ± 0.6
	5	113.9 ± 1.0
Cu^2+^	2	43.0 ± 0.7
	5	24.9 ± 0.7
Co^2+^	2	99.6 ± 2.1
	5	113.8 ± 0.2
Hg^2+^	2	Nd
	5	Nd
Ag^+^	2	Nd
	5	Nd

Reaction conditions: 0.5 mM NADH, 2 mL 50 mM citrate–phosphate buffer (pH 6.5) with different metal ions, and 5 mM 4′-chloroacetophenone were incubated for 10 min at 35 °C before adding 20 μL purified recombinant AcCR (about 0.008 mg of the purified enzyme), and recorded the changes of absorbance for 3 min

The effect of chemical agents including metal-chelator, surfactants, and sulfhydryl reagents on the recombinant AcCR was presented in Table 2. EDTA had almost no effect on the enzyme activity of the recombinant AcCR, revealing that the recombinant AcCR was a metal-ion-independent enzyme. The metal-ion-independent enzyme was conformed to the characteristic of the vast majority of SDRs (Kavanagh et al. 2008; Persson and Kallberg 2013). The sulfhydryl reagents, *β*-mercaptoethanol and iodoacetamide, had no significant effect on the recombinant AcCR activity, suggesting that there were no essential disulfide linkages and -SH groups at the catalytic sites of the recombinant AcCR (Dako et al. 2008). Surfactants were reported to play a key role in the catalytic activity of several enzymes (Ye et al. 2009). The surfactants such as SDS, Tween-80, and Triton X-100 had obviously inhibitory effect on the activity of the recombinant AcCR, which could be due to fact that the surfactants partially disrupted the hydrophobic interactions and increased the internal repulsive forces (Adler et al. 2000).

**Table 2:** Effects of chemical agents on the recombinant AcCR

Reagent	Concentration (mM)	Relative activity (%)
Control		100.0 ± 0.8
EDTA	2	95.7 ± 2.3
	5	92.4 ± 2.9
SDS	2	68.4 ± 1.2
	5	53.4 ± 1.4
*β*-Mercaptoethanol	20	94.2 ± 2.0
	40	88.1 ± 2.0
Iodoacetamide	20	97.1 ± 1.4
	40	85.3 ± 1.2
Tween-80	1% (v/v)	74.1 ± 1.9
	2% (v/v)	66.9 ± 1.8
Triton X-100	1% (v/v)	86.8 ± 1.9
	2% (v/v)	71.0 ± 2.0

Reaction conditions: 0.25 mM NADH, 2 mL 50 mM citrate–phosphate buffer (pH 6.5) with different chemical agents, 5 mM 4′-chloroacetophenone were incubated for 10 min at 35 °C before adding 20 μL purified recombinant AcCR (about 0.008 mg of the purified enzyme), and the changes of absorbance for 3 min were recorded

#### Substrate specificity and bioconversion of various carbonyl compounds

**Scheme 1:**
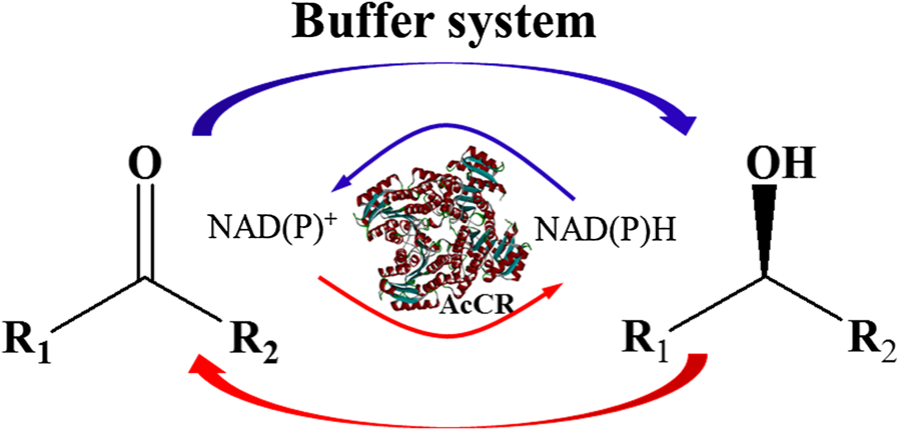
The general procedure of AcCR to conduct the oxidoreduction reaction with NAD(P)(H)

Most carbonyl reductases can catalyze reversible reaction mediated by different coenzymes, and the substrate specificity and stereoselectivity are different towards certain carbonyl reductase (He et al. 2014; Zhang et al. 2015; Takeuchi et al. 2015; Tang et al. 2014). Therefore, it was of great interest to investigate the capability of the recombinant AcCR for catalyzing redox reaction, substrate specificity, and stereoselectivity. Seventeen of prochiral carbonyl compounds and their corresponding alcohols were explored using NADH or NAD^+^ as coenzyme (Scheme 1), and the results are shown in Table 3. Glancing at the results, we found that the AcCR could not only catalyze the reduction of carbonyl groups but also could complete the dehydrogenization of the partial tested alcohols. The activities of ketones reduction were higher than those of oxidation of alcohols obviously, which was beneficial to proceed ketones reduction. The oxidation of ethyl (*R*)-(+)-4-chloro-3-hydroxybutyrate or ethyl (*S*)-(-)-4-chloro-3-hydroxybutyrate was investigated, respectively, and no oxidative activity was tested. But when ethyl-4-chloroacetoacetate was used as substrate, the relative activity was tested to be 120.6%, indicating that the reaction was not reversible towards ethyl-4-chloroacetoacetate and ethyl (4-chloro-3-hydroxybutyrate. In addition, using 2-(*R*)-octanol or 2-(*S*)-octanol as substrate, the relative activity of AcCR was far from each other (107.7% vs 2.9%), which attested that the AcCR also had stereoselectivity on the oxidation of enantiomer alcohols substrates.

**Table 3:** The relative activity of the recombinant AcCR to catalyze the oxidation of alcohols and reduction of ketones

Substrate	Structure	Relative activity (%)	Substrate	Structure	Relative activity (%)
Isopropanol		45.4			
1-Phenethyl alcohol		Nd	Acetophenone		9.2
1-(4-Methylphenyl)ethanol		16.6	4-Methylacetophenone		16.5
2-Methoxyphenethyl alcohol		1.9	2-Methoxyacetophenone		3.1
3-Methoxyphenethyl alcohol		7.7	3-Methoxyacetophenone		10.7
4-Methoxyphenethyl alcohol		11.6	4-Methoxyacetophenone		5.1
3,3-Dimethyl-2-butanol		36.5	3,3-dimethyl-2-butanone		47.5
1-(Trimethylsilyl)-ethanol		Nd	1-(Trimethylsilyl)ethanone		156.5
4-(Trimethylsilyl)-3-butyn-2-ol		2.9	4-(Trimethylsilyl)-3-butyn-2-one		114.4
Methyl 3-hydroxybutyrate		13.1	Methyl acetoacetate		81.1
Ethyl 3-hydroxybutyrate		6.2	Ethyl acetoacetate		52.1
Ethyl (*R*)-4-chloro-3-hydroxybutyrate		Nd	Ethyl-4-chloroacetoacetate		120.6
Ethyl (*S*)-4-chloro-3-hydroxybutyrate		Nd			
1-(4-Fluorophenyl)ethanol		55.1	4-Fluoroacetophenone		85.1
1-(4-Chlorophenyl)ethanol		52.3	4-Chloroacetophenone		100.0
1-(4-Bromophenzyl) alcohol		44.2	4-Bromoacetophenone		117.1
1-(4-Nitrophenzyl) alcohol		49.6	4-Nitroacetophenone		98.7
2-Pentanol		98.6	2-pentanone		158.6
2- (*R*)-Octanol		107.7	2-Octanone		87.58
2- (*S*)-Octanol		2.9			
Ethyl 2-hydroxy-4-phenylbutyrate		2.3	Ethyl 2- oxo-4-phenylbutyrate		35.8

Reaction conditions: for oxidation reaction (a) 0.5 mM NAD^+^, 2 mL 50 mM Tris–HCl buffer (pH 8.5), 50 mM alcohol compound incubated at 45 °C for 5 min before adding 20 μL purified recombinant AcCR (about 0.008 mg the purified enzyme), recorded the changes of absorbance for 3 min at 45 °C; for reduction reaction (b) 0.25 mM NADH, 2 mL 50 mM citrate–phosphate buffer (pH 6.5), 50 mM carbonyl compound at 35 °C for 5 min before adding 20 μL purified recombinant AcCR (about 0.008 mg the purified enzyme) recorded the changes of absorbance for 3 min at 35 °C

Chiral alcohols are one of the most useful building blocks for the preparation of pharmaceuticals. As the excellent relative activity of the recombinant AcCR towards those prochiral carbonyl-group substrates, it is of great interest to investigate the product yields and enantiomeric excess (*e.e.*) of the asymmetric reduction of the carbonyl compounds catalyzed by the recombinant AcCR. As shown in Table 4, AcCR exhibits a broad substrate spectrum and the enantioselectivity of this enzyme follows anti-Prelog rule which are relatively rare in nature (Tang et al. 2014; Li et al. 2015). The results shown in Tables 3 and 4 indicated that the relative activity was affected by the position of the substituents, such as the 2′-, 3′- and 4′-positions substituted acetophenone, 2′-methoxyacetophenone (16.5%), 3′-methoxyacetophenone (3.1%), and 4′-methoxyacetophenone (10.7%). High product yield and excellent enantioselectivity (>99%) were achieved when the 4′-position of acetophenone was substituted by electron-withdrawing groups such as –F, –Br, and –NO_3_, whereas the electron-releasing groups like -methyl had a negative effect on the activity of the recombinant AcCR. Moreover, it is noteworthy that the AcCR was more active to ethyl 4′-chloroacetoacetate among the investigated α-ketoesters, and the relative activity and yield were over 120.6 and 92.1%, respectively. The recombinant AcCR exhibited 114.4% relative activity to the 4-(trimethylsilyl)-3-butyn-2-one, and the product *e.e.* was over 99%, but the yield was only 62.5%, possibly because the substrate could be easily decomposed into a carbonyl alkyne and trimethyl hydroxysilane in buffer system with pH over 6.0 (Zhang et al. 2008). The product yield of reducing 3,3-dimethyl-2-butanone to 3,3-dimethyl-2-butanol was 61.7% and the product *e.e* was 80.7%, and if further reaction continued, the product *e.e* decreased markedly. What is more, unlike the acetophenone reduced to corresponding (*R*)-products, ethyl 4′-chloroacetoacetate and ethyl 2-oxo-4-phenylbutyrate were catalyzed to ethyl (*S*)-4-chloro-3-hydroxybutyrate and (*S*)-2-hydroxy-4-phenylbutyrate, respectively, which accorded with the anti-Prelog rule that the Cahn-Ingold-Prelog priority system was conversely exhibited when the substituted group contained higher priority atoms at its chiral center (Itoh 2014).

**Table 4:** Asymmetric reduction of prochiral carbonyl compounds catalyzed by the recombinant AcCR

Substrates	Products	Reaction time (h)	Con.^a^ Yield (%)	Yield (%)	*e.e.* (%)	Config.^b^
Acetophenone	Phenethyl alcohol	3	48.2	45	>99	R
2′-Methoxyacetophenone	2-Methoxyphenethyl ethanol	12	17.1	10.8	>99	R
3′-Methoxyacetophenone	3-Methoxyphenethyl ethanol	7	67.5	61.6	>99	R
4′-Methoxyacetophenone	4-Methoxyphenethyl ethanol	12	56.1	50.8	>99	R
4′-Methylacetophenone	1-(4-Methylphenyl) ethanol	3	62.8	57.7	>99	R
4′-Fluoroacetophenone	1-(4-Fluorophenyl) ethanol	7	99.1	95.6	>99	R
4′-Chloroacetophenone	1-(4-Chlorophenyl) ethanol	7	99.7	99.4	>99	R
4′-Bromoacetophenone	1-(4-Bromophenyl) ethanol	7	99.5	99.1	>99	R
4′-Nitroacetophenone	1-(4-Nitrophenyl) ethanol	7	99.8	98.6	>99	R
Methyl acetoacetate	Methyl 3-hydroxybutyrate	3	98.7	94.3	>99	R
Ethyl acetoacetate	Ethyl 3-hydroxybutyrate	3	91.7	88.5	97.6	R
Ethyl 4′-chloroacetoacetate	Ethyl 4-chloro-3-hydroxybutanoate	5	95.3	92.1	>99	S
Ethyl 2-oxo-4-phenylbutyrate	Ethyl 2-hydroxy-4-phenylbutyrate	3	72.9	68.4	83.1	S
4-(Trimethylsilyl )-3-butyn-2-one	4-(Trimethylsilyl )-3-butyn-2-ol	7	89.2	62.5	>99	R
3,3-dimethyl-2-butanone	3,3-dimethyl-2-butanol	7	76.5	61.7	80.7	R
2-Octanone	2-Octanol	3	71.6	66.9	>99	R

^a^Conversion

^b^Configuration

Reaction conditions: 4 mL citrate–phosphate buffer (50 mM, pH 6.5) containing 50 mM substrate, 0.1 mM NADH, 150 mM isopropanol, 8 U recombinant AcCR, 35 °C, 180 rpm

### Kinetic parameters assays

The kinetic constants of the purified AcCR were calculated by fitting data by linear regression to a Lineweaver–Burk double reciprocal plot. All the kinetic constants for 4′-chloroacetophenone, isopropanol, NAD(P)H, and NAD(P)^+^ are presented in Table 5. The results for reduction of 4′-chloroacetophenone showed that the enzyme had a much higher affinity for NADH than NADPH. Many carbonyl reductases from microorganism preferred NADPH much more, such as ADHs from *Lactobacillus* (Weckbecker and Hummel 2009; Leuchs and Greiner 2011), carbonyl reductase from *Gluconobacter oxydans* (Chen et al. 2015), and aldo–keto reductases from *Candida parapsilosis* (Guo et al. 2014). The NADH as coenzyme for carbonyl reductase was heavily favored for its economy and stability (Leuchs and Greiner 2011). The results for the oxidation of isopropanol (*Km* value 0.38 and 23.8 Vs 1.39 and 54.1) gave a proof that the AcCR was more affiliative to NAD^+^ than NADP^+^ when conducting the oxidation. Above all, the AcCR was an NAD(H) and NADP(H) dependent oxidordeuctase and more preferred NAD(H) as coenzyme in the redox reaction.

**Table 5:** Kinetic parameters of different substrates for the recombinant AcCR

Substrate	*K*_m_ (mM)	*V*_max_ (μmol min^−1^ mg^−1^)	*k*_cat_ (min^−1^)
NADH	0.16	5.19	145.7
4′-Chloroacetophenone	0.26		
NADPH	0.44	0.66	17.71
4′-Chloroacetophenone	2.75		
NAD^+^	0.38	11.8	265.8
Isopropanol	23.8		
NADP^+^	1.39	1.32	35.94
Isopropanol	54.1		

## Conclusions

The anti-Prelog carbonyl reductase AcCR from *Acetobacter* sp. CCTCC M209061 was cloned and its polypeptide sequence had been predicted and analyzed to confirm that the AcCR belongs to SDRs superfamily. Then AcCR was expressed heterologously, purified, and characterized. The purified enzyme preferred the inexpensive coenzyme NADH as specific electron donor. The recombinant AcCR exhibited excellent enantioselectivity, broad substrate spectrum, and highly stereoselective anti-Prelog reduction of prochiral ketones. These results suggest that AcCR is a powerful chiral tool for the production of anti-Prelog alcohols.

## Abbreviations

CR: carbonyl reductase
ADH: alcohol dehydrogenase
SDR: short-chain dehydrogenase/reductase
AcCR: carbonyl reductase from *Acetobacter* sp. CCTCC M209061
GC: gas chromatography
IPTG: isopropyl-*β*-d-thiogalactoside
